# Unusual presentation in amyloidosis

**DOI:** 10.1002/ccr3.6120

**Published:** 2022-08-03

**Authors:** Ines Mahmoud, Leila Rouached, Meriem Chammakhi, Aicha Ben Tekaya, Selma Bouden, Aycha Ben Miled, Rawdha Tekaya, Olfa Saidane, Leila Abdelmoula

**Affiliations:** ^1^ Department of Rheumatology Charles Nicolle Hospital Tunis Tunisia; ^2^ Department of Radiology Charles Nicolle Hospital Tunis Tunisia

**Keywords:** amylois, arthropathy, biopsy, multiple myeloma, spine

## Abstract

We report a case of osteolytic polyarthritis and atlanto‐axial lesion revealing multiple myeloma‐associated amyloid arthropathy in a 56‐year‐old man. Only histological exam of the salivary gland biopsy demonstrating the presence of amyloid deposits stained with congo red could confirm the diagnosis.

## Graphical abstract

We report an unusual case of multiple myeloma‐associated amyloid arthropathy revealed by a osteolytic polyarthritis and atlanto‐axial lesion.

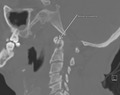

## 1 BACKGROUND

Amyloidosis is a group of diseases that are a consequence of abnormal protein deposits in various tissues of the body. Amyloid deposits can occur in a variety of organs, with involvement of the heart, kidney, gastrointestinal tract, and nervous system. Musculoskeletal manifestations of amyloidosis include myopathy, arthropathy, and osteopathy.^1^ Clinically, amyloid arthropathy (or MAA) can be mimicked by (seronegative) rheumatoid arthritis (RA). In fact, recent systematic analysis of 101 cases of MAA analyzing the spectrum of its clinical presentations revealed that MAA is more diverse than originally thought, that it is often clinically mistaken for RA, and only apparent when a tissue biopsy is carried out.^2^ The cervical spine damage, especially the atlantoaxial lesion which is common in RA, was exceptionally mentioned during amyloidosis.^3^ Solitary amyloidomas affecting the vertebrae are exceedingly rare. To date, only eight cases of primary solitary amyloidoma affecting the cervical spine have been reported in a review of literature.^4^

This rare location is of interest because of its functional and vital impact. We report here a case of osteolytic polyarthritis and atlantoaxial lesion revealing AL amyloidosis associated with multiple myeloma.

Written patient’s consent was obtained for publication.

## 2 CASE REPORT

A 56‐year‐old man with non‐insulin‐dependent diabetes (for 7 years) and kidney failure presented with worsening swelling and pain in both wrists, small joints of the hands, elbows, shoulders, and knees since 4 months. He had been on regular hemodialysis for 5 months. He also noted fatigue, weight loss, and loss of appetite. He complained of painful numbness and weakness in the legs. He had been unable to walk independently for about 1 month.

On admission, he was astenic. Physical examination showed an active limitation in shoulders, symmetrical swelling with tenderness in knees, and multiple joints of extremities, particularly in wrists, metacarpo‐phalangeal (MCP), and proximal interphalangeal (PIP) joints. There were no abnormal findings in his chest or abdomen suggestive of involvement of visceral organs. Neurologic examination revealed normal mental status and cranial nerves. He had weakness in distal limbs. He was areflexic. Sensation was absent to all modalities in the lower extremities. Passive movement of the neck was painful.

Laboratory analysis showed anemia microcytic and hypochromic (hemoglobin: 7.4 g/dl, MCV: 79 μ^3^, MCH: 68%), hypercalcemia (total calcium: 3.4 mmol/L), elevated ESR (74 mm), CRP (25 mg/L), and hypogammaglobulinemia (1.8 g/L). Serum immunofixation electrophoresis identified an Ig G Kappa‐type monoclonal gammapathy. Examination of a bone marrow aspirate found 60% of dystrophic plasma cells.

Rheumatoid factor, anti‐citrullinated peptides, and antinuclear antibodies screening turned out to be negative.

Radiographs of the hands and wrists showed nonspecific soft tissue swelling. There were no bony erosions and no evidence of calcium pyrophosphate deposition.

An ultrasound examination of the shoulders depicted synovial thickening of the tendon sheaths and of the subacromial‐sub‐deltoidian bursa, seat of scattered punctuate calcifications. Doppler signal was absent. The same changes were found at the first MCP joints of both hands.

An amyloid arthropathy was highly suspected. A minor salivary gland biopsy contained amorphous fibrillar material that stained with Congo red. Immunohistochemical exam stained the presence of kappa light chains.

**FIGURE 1:**
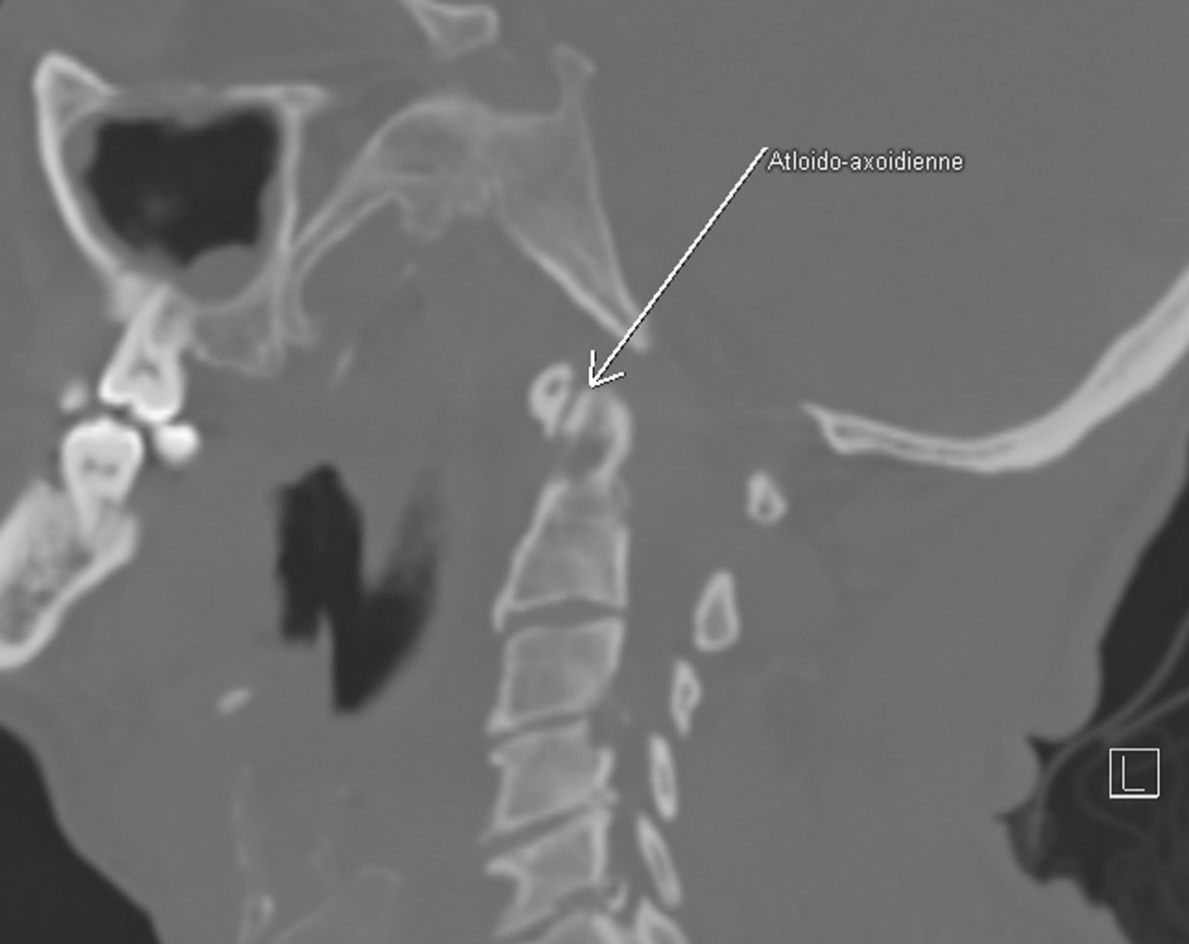
Osteolysis in synovial fold zone of the atlantoaxial articulation in CT scan

**FIGURE 2:**
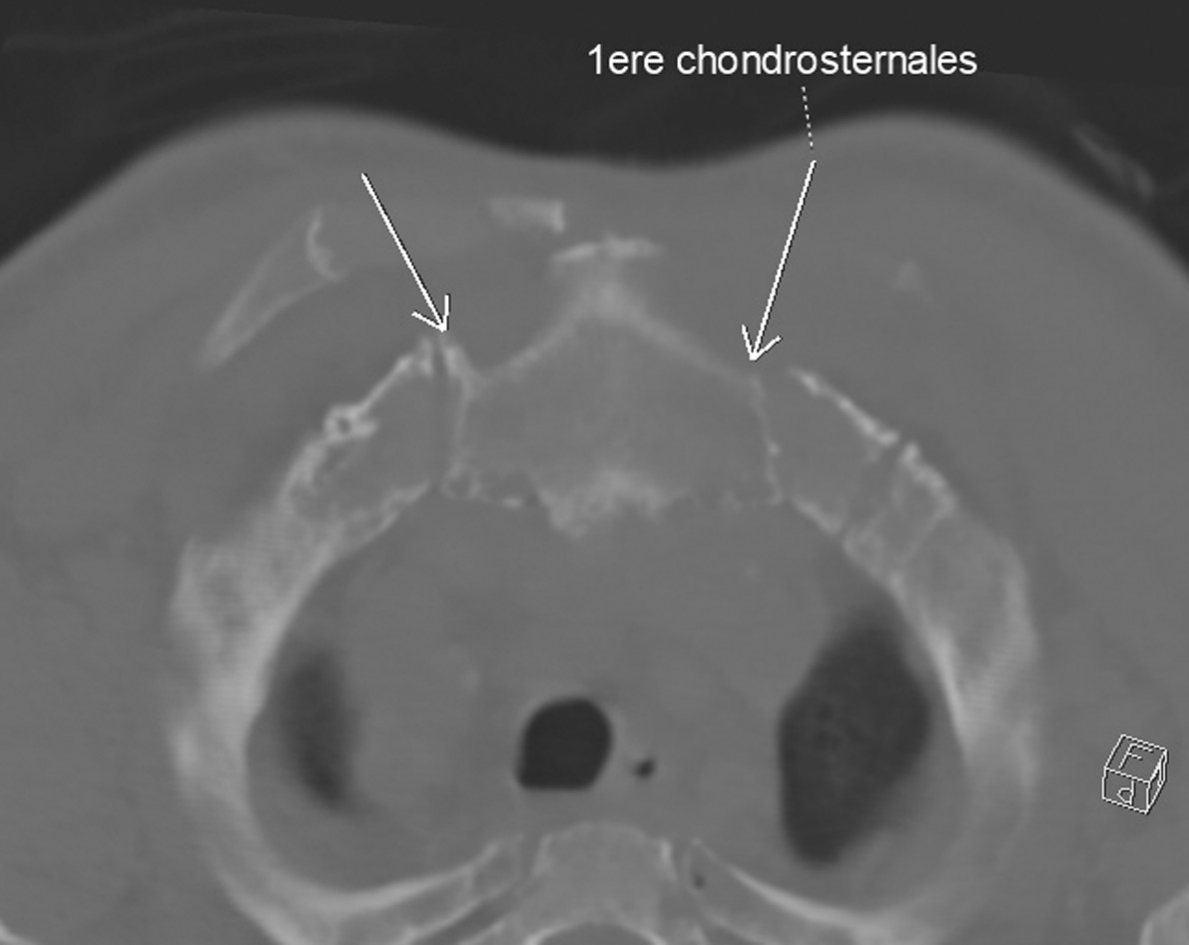
Multifocal amyloid arthropathy in the sternoclavicular joints in CT scan

A CT scan was performed demonstrating osteolysis in synovial fold zone of the atlantoaxial articulation (Figure 1), the two sternoclavicular and the right acromioclavicular joints, each associated with periarticular soft tissues mass syndrome compatible with multifocal amyloid arthropathy (Figure 2).

Echocardiogram shows a good function of the left ventricle and no signs of cardiac amyloidisis.

Electromyography shows an axonal polyneuropathy, involving sensory more than motor fibers of feet and proximal legs.

These findings led to a diagnosis of Kappa‐light chain myeloma and AL amyloidosis with kidney, neurologic, and joint involvement.

One week later, the patient developed septicaemia due to *Staphylococcus aureus* and unfortunately died before treatment initiation.

## 3 DISCUSSION

Amyloid arthropathy is nowadays a well‐recognized entity.^1^ Amyloidosis encompasses a spectrum of diseases characterized by extracellular deposition of amyloid, an insoluble proteinaceous material with a beta‐pleated sheet configuration.^4^ It may include joints, joint capsule, or articular cartilage.

On light microscopic examination, amyloid appears as an eosinophilic amorphous hyaline extracellular substance, which turns yellow‐green after Congo Red staining and examination under polarized light.^4^ These histopathologic stainings are necessary to differentiate amyloid from other hyaline deposits, such as collagen.

Among the amyloidoses, arthropathy preferentially occurs in the β2‐microglobulin‐derived one, which complicates chronic renal failure with long‐term hemodialysis. It can occur also in AL amyloidosis associated with multiple myeloma. In the majority of cases, multiple myeloma‐associated amyloid arthropathy (MAA) is a revealing sign of this monoclonal gammapathy.^2^

It is most commonly described as a seronegative nonerosive polyarthritis, mimicking RA.^5^, ^6^ Indeed, MAA may present clinically with symmetrical swelling and tenderness in multiple joints: shoulders, wrists, knees, and metcarpophalangeal joints. Weight loss and asthenia are frequently present too. Our patient showed symmetrical swelling of knees, wrists, MCP, PIP, and both shoulders, the so‐called “shoulder‐pad sign”.^7^ This sign is resulting from periarticular soft tissue amyloid deposition and is essentially pathognomonic for immunoglobulin AL amyloidosis.

Ultrasonography could be a helpful tool in the diagnosis. The findings include thickening of supraspinatus and biceps tendons, synovial thickening, the presence of hypoechoic masses around tendons, bursas synovial enlargement, and effusion within the bursa. The power Doppler signal is always negative.^8^

Radiologically, counter to RA, MAA is rarely erosive. The classical X‐ray is usually normal. The joint space is preserved or even widened. Some bone cysts may be encountered mainly at the carpus, inferior extremity of the radius, superior extremity of the humerus, or the femoral neck.^9^, ^10^

The atlantoaxial articulation may also be affected. Our patient complained about neck pain and stiffness. The cervical scan showed an osteolysis of the odontoid process. Only one case of amyloid C1–C2 synovitis with subluxation of atlantoaxial joint was reported by Fautrel et al.^3^ in their cohort of 311 patients. In fact, the spine may be involved in general amyloidosis but also in localized one. Primary amyloidoma of the spine is very rare, with fewer than 25 cases reported in the literature.^4^ The lesion usually arises from within the bone marrow of the vertebral body without involving the disc space. Based on available case reports, primary spinal amyloidoma has a predilection for the thoracic region.^4^ Previously reported cases of cervical primary amyloidosis are summarized in the review of litterature of Brian et al.^4^

Once the cervical spine affected, symptoms may vary from common cervicalgia to spinal cord compression. Usually, magnetic resonance imaging reveals a low signal on T1 and T2‐weighted images with a peripheral enhancement after gadolinium injection. In case of neurological complication, surgical excision completed by stabilization may be required. Outcome seems to be excellent.^11^

An osteolysis in synovial fold zone of the two sternoclavicular joints with periarticular soft tissues mass syndrome was identified in our patient. Goffin et al found amyloid deposits in the sternoclavicular fibrocartilage.^12^

## 4 CONCLUSION

Myeloma‐associated amyloid arthropathy should be one of the differential diagnoses of RA. Both MAA and RA can be responsible of symmetrical erosive polyarthritis, cervical spine, and sternoclavicular lesions. In this case, only the histological exam of the biopsied tissues demonstrating the presence of amyloid deposits stained with congo red can confirm the diagnosis.^13^, ^14^

## AUTHOR CONTRIBUTIONS

Ines Mahmoud: Writing. Leila Rouached: Writing. Meriem Chammakhi: collecting information. Aicha Ben Tekaya: writing. Selma Bouden: analyses. Aycha Ben Miled: interpretation of figures. Rawdha Tekaya: collecting information. Olfa Saidane: correction. Leila Abdelmoula: supervision.

## CONFLICT OF INTEREST

The authors have no conflict of interest to declare.

## ETHICAL APPROVAL

Our case report complies with the Declaration of Helsinki.

## CONSENT

Informed consent has been obtained from the patient.

## Data Availability

None.
